# Comparison of Early Clinical Results for Femoral Neck System and Cannulated Screws in the Treatment of Unstable Femoral Neck Fractures

**DOI:** 10.1111/os.13098

**Published:** 2021-08-05

**Authors:** Xiao‐qiang Zhou, Zhi‐qiang Li, Ren‐jie Xu, Yuan‐shi She, Xiang‐xin Zhang, Guang‐xiang Chen, Xiao Yu

**Affiliations:** ^1^ Department of Orthopaedics The Affiliated Suzhou Hospital of Nanjing Medical University, Suzhou Municipal Hospital, Gusu School, Nanjing Medical University 26# Daoqian Street Suzhou Jiangsu, Province China

**Keywords:** Cannulated screw, Clinical efficacy, Femoral neck fracture, Femoral neck system, Internal fixation

## Abstract

**Objective:**

To compare early clinical effects of the femoral neck system (FNS) and three cannulated screws for the treatment of patients with unstable femoral neck fractures.

**Methods:**

A retrospective analysis with pair matching of 81 patients who received FNS or cannulated screw internal fixation for Pauwels type‐3 femoral neck fracture in our hospital from January 2019 to December 2019 was conducted. Patients who received FNS were the test group, and those who received cannulated screws comprised the control group. Matching requirements were as follows: same sex, similar age, and similar body mass index (BMI). A total of 30 pairs were successfully matched at a 1:1 ratio, including 12 males and 18 females. The average age of the patients in the FNS group was 54.53 ± 6.71 years. In the cannulated screw group, the average age of the patients was 53.14 ± 7.19 years. The operation time, intraoperative blood loss, hospital stay, hospitalization cost, postoperative visual analog scale (VAS) score, time to walking without crutches, Harris score, femoral head necrosis rate, and complication rate were compared between the groups.

**Results:**

Postoperative re‐examination of radiographs showed satisfactory reduction in all patients, and all patients were followed up for 10–22 months. Those in the FNS group had lower postoperative VAS scores, earlier times to walking without crutches, higher Harris scores at the last follow‐up, and lower complication rates (*P* < 0.05). VAS scores were lower in the FNS group (3.13 ± 1.07 scores) than in the cannulated screw group (3.77 ± 1.04 scores) (*P* = 0.018). Patients in the FNS group (5.23 ± 1.33 months) recovered to walking without crutches earlier than did those in the cannulated screw group (6.03 ± 1.45 months) (*P*<0.001). In addition, a statistically higher postoperative Harris score was detected in the FNS group (86.16 ± 7.26) than in the cannulated screw group (82.37 ± 7.52) (*P* = 0.039). Overall, a higher incidence of complications was observed in the cannulated screw group (9/30) than in the FNS group (2/30) (*P* = 0.042). However, intraoperative blood loss and hospitalization costs were greater in the FNS group (*P* < 0.05). Intraoperative blood loss was greater in the FNS group (99.73 ± 4.69) than in the cannulated screw group (30.27 ± 9.04) (*P*<0.001). In addition, patients in the FNS group (46976 ± 2270 ¥) spent more on hospitalization costs than did those in the cannulated screw group (15626 ± 1732 ¥) (*P*<0.001). No statistically significant difference in operation time, hospital stay, or femoral head necrosis rate was observed between the two groups (*P* > 0.05).

**Conclusion:**

For patients with unstable femoral neck fractures, FNS has better clinical efficacy than cannulated screws, though it is also more expensive.

## Graphical abstract

To compare early clinical effects of the femoral neck system (FNS) and three cannulated screws for the treatment of patients with unstable femoral neck fractures. We designed a retrospective analysis with pair matching. This study found that compared with cannulated screws, FNS is a suitable option for the treatment of Pauwels type‐3 femoral neck fractures. This approach is characterized by its accurate efficacy, simple procedure, reduced trauma, faster recovery, and fewer complications, though it is more expensive. FNS's excellent biomechanical performance and clinical efficacy make it a new choice for the treatment of unstable femoral neck fractures.

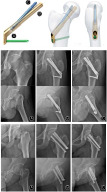

## Introduction

With the aging of the population, the incidence of hip fractures is on the rise. Femoral neck fractures are the most frequent hip fractures and often caused by high‐energy trauma in young adults. The annual cost of treating femoral neck fractures is huge, and it will increase in the future. The treatment of femoral neck fracture should take into consideration fracture types and individualized characteristics, aiming to minimize adverse consequences. For patients with femoral neck fracture who are younger than 65 years old, internal fixation with femoral head preservation is considered the first choice of treatment^1^. Due to the blood supply of the femoral head and special morphological anatomy, the overall incidence of femoral head necrosis after femoral neck fracture remains high. This situation seriously damages quality of life.

At present, incidences of fracture nonunion and necrosis of femoral head sharply decrease owing to the advanced progress made on closed reduction and minimally invasive internal fixation. Compared with hip arthroplasty, internal fixation has advantages of less trauma, short operation time, less bleeding, and low cost, and it is consistent with the concept of hip preservation. Several surgical implants have been used widely for the treatment of femoral neck fracture in young patients, including the proximal locking plate, dynamic hip system, sliding hip screw and cannulated screw. Nevertheless, the proportion of revision surgery remains high. The inadequacy of biomechanics to maintain sufficient stability during fracture healing is the main reason for this high failure rate. Many implants can be used for internal fixation of femoral neck fractures, but the failure rate is not much different. Current opinion remains inconsistent with regard to the selection of internal fixation approaches to achieve better outcomes of fracture end stability, bone union, decreasing incidences of femoral head necrosis, and complications^2^. Different internal fixation devices have their own advantages and disadvantages, and their efficacy on the treatment of different types of fractures remains controversial.

To date, the use of 7.5‐mm cannulated screws (Triad) is the most commonly approach for internal fixation. By compressing the fracture end, fixation with three cannulated screws contributes to anatomical reduction, rigid internal fixation, and union of femoral neck fractures^3^. However, some problems in the fixation of partially unstable femoral neck fractures with three cannulated screws remain^4^, ^5^. Although the design of cannulated screw can provide relatively large pulling force, due to its dynamic compression characteristics, it cannot provide enough support for the femoral neck and prolong the postoperative healing time. If the fracture end is absorbed or a strong vertical shear force is generated against a vertical femoral neck fracture, the internal fixation is likely to become loose. Increasing fracture angle in unstable femoral neck fractures may result in a higher rate of fixation failure.

An ideal minimally invasive implant guarantees stability of the internal fixation without shortening of the femoral neck or tilt or rotation of the femoral head. The healing of femoral neck fractures requires maintaining the stability of the fracture site in the coronal and sagittal planes and absolute rotational stability. Therefore, the internal fixation device should be able to keep the fracture end in contact, maintain firm stability, and be able to resist daily stress, so as to ensure the healing of the fracture.

**Fig. 1:**
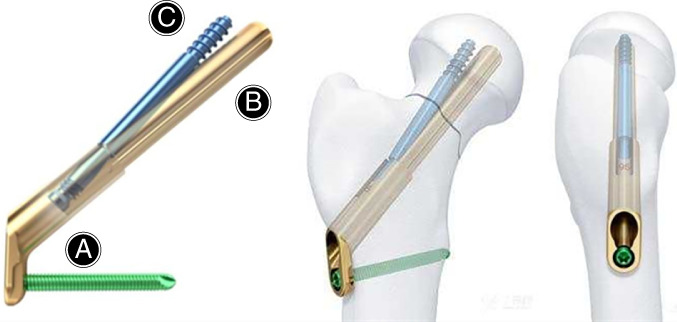
FNS consists of three parts: (A) the plate and locking screw in the angular stable structure (neck‐shaft angle = 130°, size of the locking screw = 5.0 mm); (B) a round, blunt‐headed screw bolt (diameter = 10 mm, length = 75–130 mm); (C) a round, blunt‐headed anti‐rotation screw that can be locked to the screw bolt (diameter = 6.4 mm).

For dynamically fixing fractured femoral necks, a novel femoral neck system (FNS) was developed by the Lower Extremity Expert Group, the Association for the Study of Internal Fixation and DePuy Synthes Products (Fig. 1). FNS is able to provide angular stability with minimally invasive procedures. An implant with a small side plate can be fixed to the femoral shaft, which reduces the footprint of the implant and at the same time fixes the femoral head by locking the screw in the bolt. By combining minimally invasive insertion technology with the stability of the dynamic hip screw, FNS retains the function of the femoral head as much as possible. Notably, FNS highlights the biological characteristics of fracture healing *via* compression at the fracture end. This novel implant may be a major advance in the treatment of femoral neck fracture. The indications for FNS include subcapital femoral neck fractures, transcervical femoral neck fracture, and vertical shear femoral neck fractures. Contraindications include patients with intertrochanteric femoral fractures, femoral head fractures, subtrochanteric femoral fractures, and pathological femoral neck fractures.

Overall, there is scant literature about clinical application of the newly developed FNS. Hence, the purpose of this study was: (i) to retrospectively analyze the therapeutic efficacy of FNS and cannulated screw fixation for the treatment of unstable femoral neck fracture in younger patients, hoping to provide some evidence‐based references for clinical treatment; (ii) discuss the disadvantages of cannulated screw in the treatment of Pauwels type‐3 femoral neck fracture and analyze the reasons for these disadvantages; and (iii) to explore the reasons for the better clinical efficacy of FNS.

## Materials and Methods

### Inclusion and Exclusion Criteria

Inclusion criteria: (i) patients of fresh unilateral Pauwels type‐3 femoral neck fracture; (ii) patients who received FNS or cannulated screw fixation in our department from January 2018 to December 2019; (iii) the major evaluation indicators are operation time, intraoperative blood loss, hospital stay, hospitalization cost, quality of fracture reduction, day 1 postoperative visual analog scale (VAS) score, time to walking without crutches, Harris score, complication rate, and femoral head necrosis rate; (iv) follow‐up results of the patients were recorded; and (v) a retrospective study.

Exclusion criteria: (i) excessive drinking, long‐term history of hormone drugs or femoral head necrosis; (ii) other severe diseases; (iii) pathological fractures; and (iv) severe cognitive dysfunction.

### Patients

A retrospective analysis was performed from January 2018 to December 2019 using 81 eligible femoral neck fracture patients undergoing FNS or cannulated screw fixation in the Department of Orthopedics, The Affiliated Suzhou Hospital of Nanjing Medical University, Suzhou Municipal Hospital. Among them, 30 patients were treated with FNS and 51 patients with three traditional cannulated screws. The patients treated with FNS were selected as the experimental group; those treated with cannulated screws were selected as the control group. A pair‐matched clinical research study was performed with the following requirements: sex, age ± 3 years old, body mass index (BMI) ± 2 kg/m^2^. A total of 30 pairs were successfully matched at a 1:1 ratio, including 12 males and 18 females. This trial was approved by the ethics committee of Suzhou Municipal Hospital (IEC‐C‐008‐A07‐V1.0). All the study participants provided written informed consent for the study.

### Surgery

Femoral neck fracture surgery was conducted within 48 h of admission by the same group of surgeons.

#### FNS Group

*Anesthesia and Position*. Patients received spinal anesthesia or general anesthesia and were fixed in a supine position with orthopaedic traction bed.

*Reduction and Observation*. The operated limb was placed on the traction frame in an abducted, internally rotated position. Adjust the degree of internal and external rotation and the angle of adduction according to the fracture reduction. Quality of fracture reduction was observed by C‐arm localization.

*Positioning and Fixation*. A longitudinal incision of about 4 cm was cut on the lateral hip to expose the proximal femur. A Kirschner wire was inserted into the femoral head alongside the lateral femur to temporarily fix the femoral neck fracture with satisfactory reduction.

*Internal Fixation Implant*. Under the guidance of a localizer at 130°, a guide wire was inserted into the femoral neck, which was placed in the centre of both the femoral neck and the femoral head in the anteroposterior view. If necessary, adjust the position of the guide wire according to the image of the fluoroscopic C‐arm machine. Determine length and ream for insertion, then insert implant. Drill for anti‐rotation screw after removing the guide wire, then locking the anti‐rotation screw and screw insertion. Attach multifunction rod for compression. FNS (DePuy Synthes Products, USA) was inserted after reaming and sounding. A locking screw was placed in the distal hole and rotated in the femoral neck, after which the temporarily fixed Kirschner pin was removed.

*Final Check*. Postoperative reduction and position of internal fixation were observed by C‐arm localization after instrument disassembly.

**Fig. 2:**
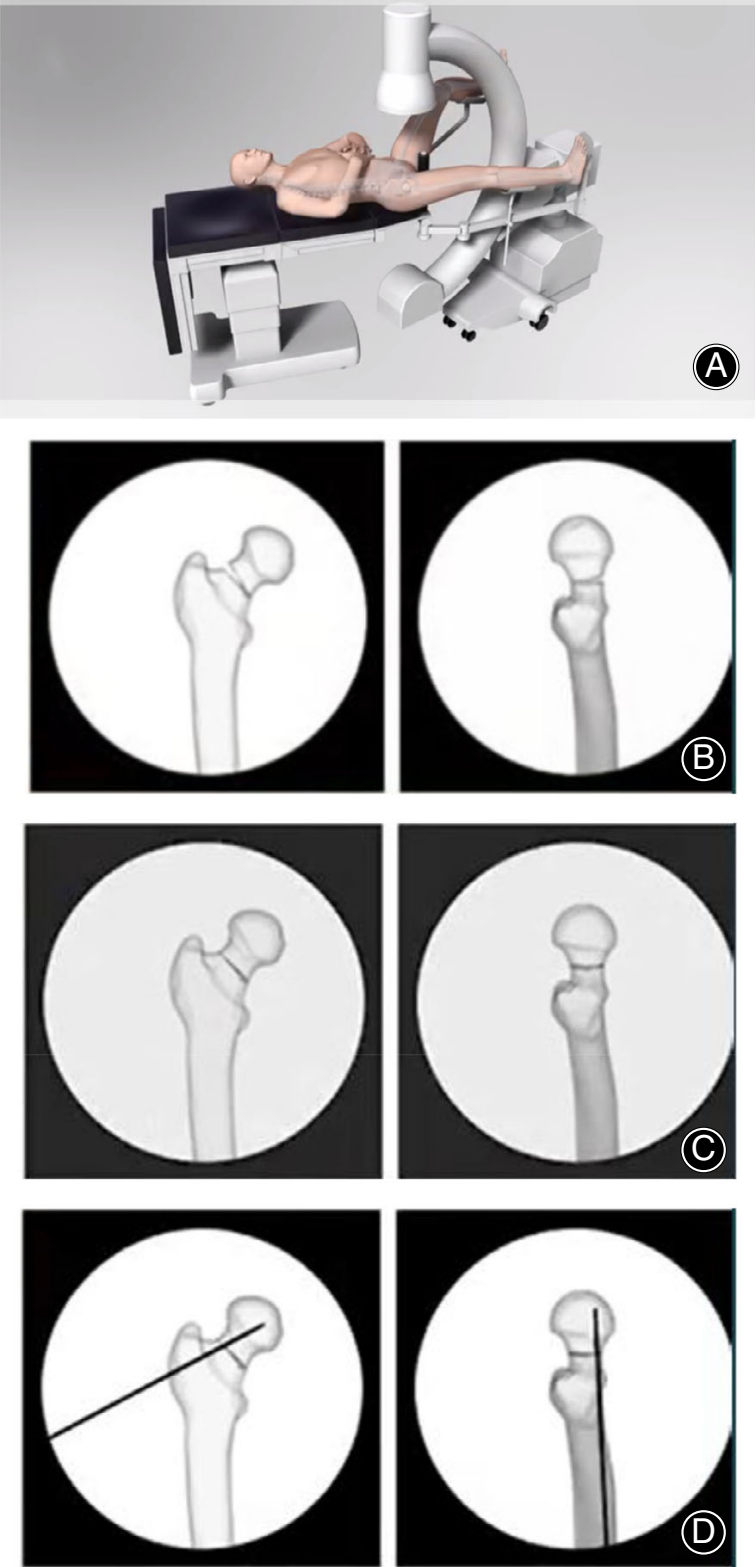
FNS surgery preparation: (A) position patient; (B) original fracture; (C) reduce fracture; (D) temporarily fix fracture.

**Fig. 3:**
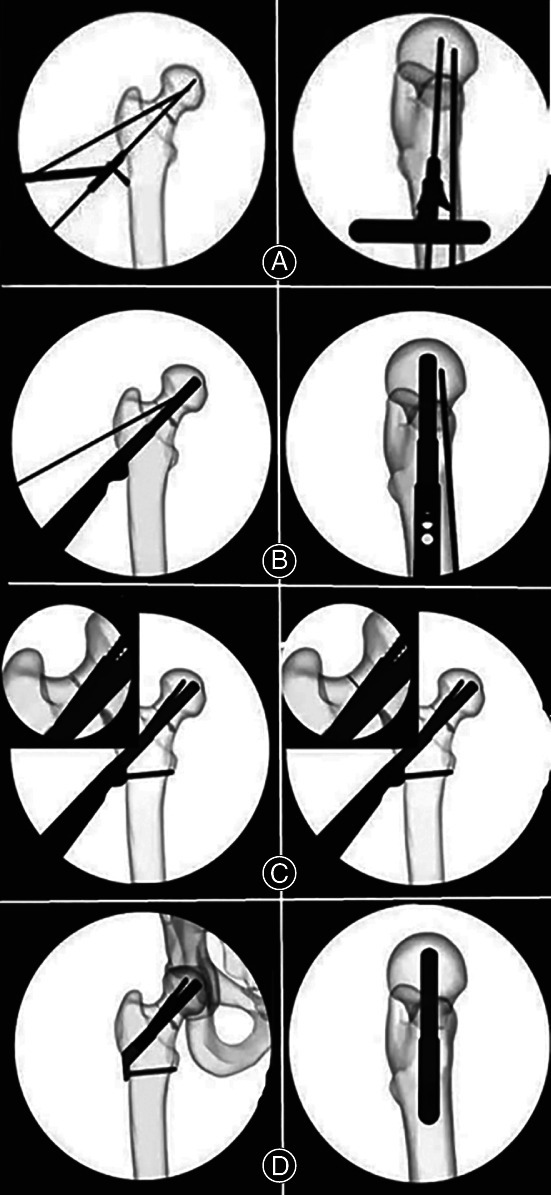
FNS implant insertion: (A) insert guide wire; (B) insert implant; (C) anti‐rotation screw and locking screw insertion, apply compression; (D) final check.

Brief steps and technical points of the surgery are depicted in Figs 2, 3.

#### Cannulated Screw Group

*Anesthesia and Position*. Patients received spinal anesthesia or general anesthesia and were fixed in a supine position.

*Reduction and Observation*. The operated limb was placed on the traction frame in an abducted, internally rotated position. Adjust the degree of internal and external rotation and the angle of adduction according to the fracture reduction. Quality of fracture reduction was observed by C‐arm localization.

*Positioning and Fixation*. For patients who received cannulated screw (DePuy Synthes, JOHNSON & JOHNSON, New Brunswick, US) fixation, three parallel guide needles were inserted into the femoral head along the longitudinal axis of the femoral neck in the shape of an inverted triangle^6^. The needles were placed in the centre of the femoral neck and the femoral head, as well as 1 cm below the articular surface.

*Internal Fixation Implant*. A 1‐cm incision was cut at the tip of the parallel guide needles. After exposing the lateral cortical bone, a hole was drifted until the fracture end. An appropriate cannulated screw was rotated under the guidance of the parallel guide needle, which was localized by X‐ray.

*Final Check*. Postoperative reduction and position of internal fixation were observed by C‐arm localization. The parallel guide needles were finally removed.

### Perioperative Management

**Fig. 4:**
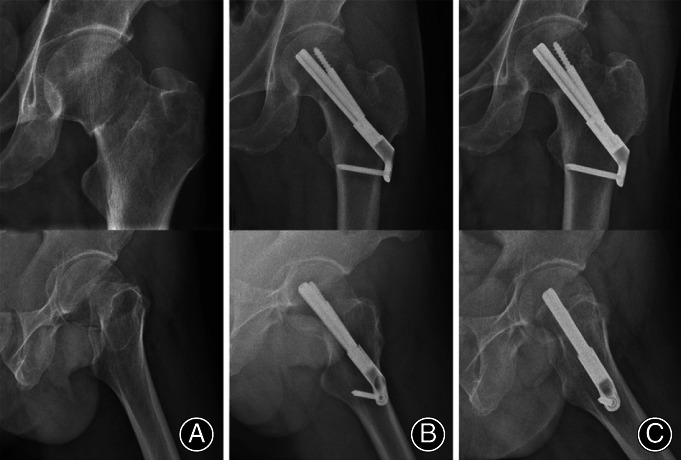
A typical case treated by FNS. A 54‐year‐old male patient with a left Pauwels type‐3 femoral neck fracture. (A) Preoperative anteroposterior view; (B) satisfactory reduction on the first day postoperatively; (C) fracture healing and stable internal fixation by the third month postoperatively.

**Fig. 5:**
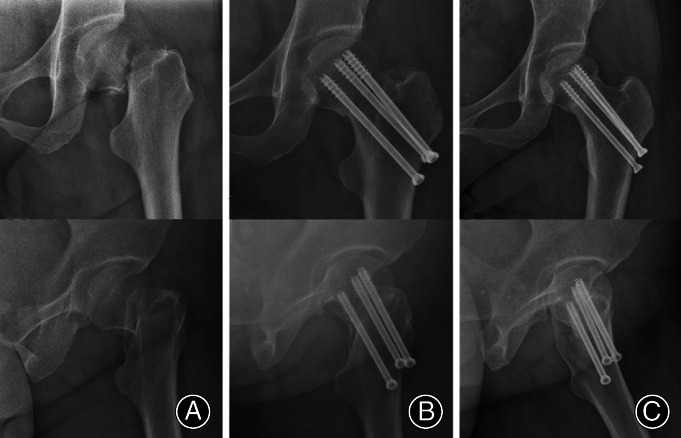
A typical case treated by cannulated screw fixation. A 52‐year‐old female patient with a left Pauwels type‐3 femoral neck fracture. (A) Preoperative anteroposterior view; (B) satisfactory reduction on day 1 postoperatively; (C) fracture healing and stable internal fixation by the third month postoperatively.

Postoperative multimodal analgesia and anti‐coagulation using rivaroxaban (Bayer Schering Pharma AG, Leverkusen, Germany) were performed. The patients were encouraged to begin exercise within 24 h of the surgery. By the second postoperative week, the patients started to exercise with the help of crutches, though weight‐bearing on the affected limb was forbidden. Partial weight‐bearing exercise was encouraged at week 6, recovering to normal exercise based on the patient's condition, followed by walking without crutches. The patients were followed up at 1, 3, 6, 9, and 12 months after the operation. Typical cases that received FNS and cannulated screw fixation are depicted in Figs 4, 5, respectively.

### Testing Indices

Operation time, intraoperative blood loss, quality of fracture reduction, day 1 postoperative VAS score, hospital stay, hospitalization cost, time walking without crutches, Harris score, complication rate, and femoral head necrosis rate were recorded.

#### Quality of Fracture Reduction

The quality of fracture reduction was assessed based on the quantitative indicators proposed by Haidukewych *et al*., as follows: (i) excellent reduction—displacement after reduction <2 mm and deformity angle at any plane <5°; (ii) fair reduction—displacement after reduction ranging from 6 to 10 mm and deformity angle at any plane ranging from 11°–20°; and (iii) poor reduction—displacement after reduction >10 mm and deformity angle at any plane >20°^7^. We confirmed it through the day 1 postoperative radiograph. This measurement indicates the quality of the operation.

#### First Day Postoperative Visual Analog Scale (VAS) Score

The visual analog scale (VAS) score can help patients assess the degree of pain. A 10‐cm horizontal line was drawn on the paper. At the end of the horizontal line are 0 and 10 score. A score of 0 means no pain, and 10 means most severe pain. For the middle scores, different numbers mean different degrees of pain. Patients can choose one number from 0 to 10 to represent their pain, according to their own feeling.

#### Time to Walking Without Crutches

At the beginning, the patients started to exercise with the help of crutches. When the X‐ray shows that the fracture has healed, walking without crutches was encouraged. Time to walking without crutches was recorded. This parameter, on the other hand, reflects the speed of postoperative fracture healing and patient recovery.

#### Harris Score

Harris score is currently the most universal hip joint function scoring system in the world. It is used to quantitatively evaluate the functional state of the hip joint before and after surgery. The main items of Harris score include pain, daily activities, deformity, range of motion. These four items total 100 points and are graded as follows: 90–100 is excellent; 80–89 is good; 70–79 is medium; and less than 70 is poor.

#### Complication Rate and Femoral Head Necrosis Rate

Complications included bone nonunion, loss of reduction, and loosening of internal fixation. X‐ray examination was performed to observe fracture healing, internal fixation, and whether there is femoral head necrosis. If the X‐ray film shows that the fracture has not healed 9 months after the operation, it is considered nonunion. Regarding loss of reduction and loosening of internal fixation, compared with postoperative X‐ray film, there were situations such as screw loosening, internal fixation entering the joint, coxa vara (>10°), fracture displacement >5 mm, shortening and displacement of the femoral neck (>10 mm), etc. during follow‐up. Computed tomography (CT) or magnetic resonance imaging (MRI) examinations were performed on patients with suspected femoral head necrosis.

### Statistical Analysis

SPSS 25.0 (IBM, Armonk, NY, USA) was used for statistical analysis. Continuous data are expressed as mean ± SD. Age, BMI, operation time, intraoperative blood loss, length of stay, hospital stay, hospitalization cost, day 1 postoperative VAS score, time to walking without crutches, and Harris score were compared using Student's *t* test. Categorical data are summarized by ratios and percentages. Sex, postoperative reduction, femoral head necrosis rate, and complication rate were compared by the χ^2^ test or Fisher's exact test. *P* < 0.05 was considered statistically significant.

## Results

### Demographic Results

The average age of the patients in the FNS group was 54.53 ± 6.71 years, with a BMI of 23.24 ± 2.12 kg/m^2^. In the cannulated screw group, the average age of the patients was 53.14 ± 7.19 years, and the BMI was 22.73 ± 2.13 kg/m^2^. No significant differences in preoperative baseline characteristics were identified (*P* > 0.05).

### Quality of Fracture Reduction

All patients achieved satisfactory postoperative reduction. In the FNS group and the cannulated screw group, 24 and 25 patients achieved an excellent reduction, and six and five achieved a fair reduction, respectively, with no significant difference (*P* = 0.739).

### Surgery and Hospitalization Indices

To investigate surgery and hospitalization indices in each group, we compared operation time, intraoperative blood loss, hospital stay, and hospitalization cost between the FNS group and the cannulated screw group (Table 1). All patients were followed up for 10–22 months. Intraoperative blood loss was greater in the FNS group (99.73 ± 4.69) than in the cannulated screw group (30.27 ± 9.04) (*P*<0.001). In addition, patients in the FNS group (46976 ± 2270 ¥) spent more on hospitalization costs than those in the cannulated screw group (15626 ± 1732 ¥) (*P*<0.001). No significant differences in operation time or hospital stay were detected between the FNS group and the cannulated screw group (*P* > 0.05). Therefore, the FNS group had similar operation time and hospital stay to the cannulated screw group, but intraoperative blood loss and hospitalization costs were more in the FNS group.

**TABLE 1:** Operation time, intraoperative blood loss, hospital stay, and hospitalization cost between the FNS group and cannulated screw group (mean ± SD)

Groups	Operation time (min)	Intraoperative blood loss (mL)	Hospital stay (days)	Hospitalization cost (RMB/¥)
FNS	42.83 ± 4.69	99.73 ± 52.73	5.07 ± 1.31	46978 ± 2270
Cannulated screw	40.90 ± 5.22	30.27 ± 9.04	5.33 ± 1.52	15626 ± 1732
*t* value	1.595	4.747	1.547	54.825
*P* value	0.122	<0.001	0.133	<0.001

### Clinical Efficacy

Notably, to compare the clinical efficacy between the FNS group and the cannulated screw group, VAS scores, time to walking without crutches and Harris score were analyzed (Table 2). VAS scores were lower in the FNS group (3.13 ± 1.07 scores) than in the cannulated screw group (3.77 ± 1.04 scores) (*P* = 0.018). Patients in the FNS group (5.23 ± 1.33 months) recovered to walking without crutches earlier than those in the cannulated screw group (6.03 ± 1.45 months) (*P*<0.001). In addition, a statistically higher postoperative Harris score was detected in the FNS group (86.16 ± 7.26) than in the cannulated screw group (82.37 ± 7.52) (*P* = 0.039). In summary, the FNS group performed better in VAS scores, time to walking without crutches, and Harris score.

**TABLE 2:** Postoperative VAS score, time to walking without crutches, and Harris score between the FNS and cannulated screw groups (mean ± SD)

Groups	Postoperative VAS score	Time to walking without crutches (months)	Harris score
FNS	3.13 ± 1.07	5.23 ± 1.33	86.16 ± 7.26
Cannulated screw	3.77 ± 1.04	6.03 ± 1.45	82.37 ± 7.52
*t* value	2.520	5.174	2.164
*P* value	0.018	<0.001	0.039

### Complications and the Femoral Head Necrosis

In fact, complications occurred in only two of the 30 patients in the FNS group. One patient with delayed union was conservatively treated with drugs to promote fracture healing, and the fracture was healed 7 months after the operation. One patient was treated by total hip arthroplasty after removing the internal fixation because the fracture reduction was lost after another fall. During the follow‐up period, there was no case of osteonecrosis of the femoral head. A total of nine patients developed postoperative complications in the cannulated screw group. One patient with delayed union was conservatively treated with drugs to promote fracture healing, and the fracture was healed 8 months after the operation. Two patients with delayed union were not treated conservatively and underwent total hip arthroplasty after internal fixation removal. Two patients also lost reduction, but the fractures healed; they were followed up for further observation. The fractures of three patients with internal fixation loosening healed, and the internal fixations were removed. During the follow‐up period, one patient experienced osteonecrosis of the femoral head and underwent total hip arthroplasty. Overall, a higher incidence of complications was observed in the cannulated screw group (30%) than in the FNS group (6.7%) (*P* = 0.042).

## Discussion

### Therapeutic Efficacy of FNS and Cannulated Screw Fixation

Our study recruited femoral neck fracture patients of a younger age (<65 years) who were treated with FNS or cannulated screw fixation and followed up for 10–22 months. We found that for Pauwels type‐3 unstable femoral neck fracture, FNS has significant advantages with regard to early clinical efficacy and complication rate compared with the traditional three cannulated screws and does not increase surgical trauma. The disadvantage is that the cost is relatively high.

This study found no significant difference in operation time or length of stay between patients treated with FNS and cannulated screw fixation. The average intraoperative blood loss in the FNS group was 99.73 mL. Although the difference was statistically significant, the amount of blood loss was still small and had no effect on postoperative rehabilitation or the clinical effect. The fast operation time and lack of trauma suggest that FNS treatment is a simple procedure and a good choice. FNS effectively reduces soft tissue exposure and usually only requires a lateral incision of approximately 4–5 cm; in addition, only partially cutting open the lateral vastus muscle is needed, with no damage to the gluteus medius.

Based on a fast‐track programme, all patients underwent surgery within 48 h of admission. Postoperative multimodal analgesia and anticoagulation were routinely administered. Moreover, the patients were encouraged to exercise their hip joint early, aiming to prevent postoperative thrombosis. As a result, no evidence of deep vein thrombosis or pulmonary embolism was found in this study. Some studies have reported that poor reduction following femoral neck fracture is a risk factor for complications and femoral head necrosis^8^. In our study, all patients achieved a satisfactory reduction by day 1 postoperatively, and there was no significant difference between the two groups.

### Disadvantages of Cannulated Screw in the Treatment of Pauwels Type‐3 Femoral Neck Fracture

The Pauwels angle reflects the interaction between compressive stress and shear force during the healing process of femoral neck fractures, and it has been widely used in clinical practice. As the Pauwels angle increases, the shear force acting on the fracture end gradually becomes the main force, leading to an increase in the complication rate^9^. At present, Pauwels type‐1 is considered to be a stable fracture, Pauwels type‐3 is an unstable fracture, and Pauwels type‐2 is somewhere in between. Liporace *et al*. reported that the fixed capacity of cannulated screw fixation is weaker than that of other internal fixation in the treatment of vertical femoral neck fractures; it may lead to shortening of the femoral neck and hip function damage, especially in osteoporosis patients^10^. Other evidence has shown that cannulated screw fixation is particularly suitable for the treatment of Garden I + II and Pauwels I + II femoral neck fractures^5^, ^11^, ^12^. According to our clinical experience, the effect of cannulated screws for the treatment of stable femoral neck fractures is essentially satisfactory, though there are some problems for unstable femoral neck fractures. Due to the high cost of FNS, we utilized FNS and cannulated screws in a comparative study for unstable femoral neck fractures (Pauwels type‐3). We believe that a single cannulated screw lacks angular stability, exhibiting poor capacity against shear stress. In addition, as a single screw is prone to longitudinally cut distal fractures, the incidence of complications in the treatment of Pauwels type‐3 femoral neck fractures is high.

### Reasons for the Better Clinical Efficacy of FNS

Moreover, FNS resulted in better biomechanical properties than conventional internal fixation using three cannulated screws for the treatment of Pauwels type‐3 femoral neck fracture. The biomechanical advantages of FNS have been previously reported. Karl *et al*. compared the axial compression load and cycle times for shortening the femoral neck by 15 mm in biomechanical testing with a 0.1‐N increase per cycle^13^. These authors pointed out that FNS presents better overall structural stability than internal fixation using three cannulated screws. FNS involves not only angular stability but also rotational stability. Schopper *et al*. further demonstrated that FNS leads to higher stability and better resistance to varus and deformation in femoral neck fractures than Hansson Pins^14^. Xu *et al*. used FNS to treat 16 patients with femoral neck fracture, including seven cases of Garden type‐3 and six cases of Garden type‐4. The results were satisfactory at the last follow‐up, and none of the patients experienced complications such as internal fixation loosening^15^.

We believe that the pronounced efficacy in femoral head fracture patients might be explained by the better biomechanical performance of FNS. At the last follow‐up in our study, the Harris score was significantly higher, and the incidence of complications lower in the FNS group. Overall, the combination of FNS bolts with anti‐rotation screws avoids the “Z” effect on the cutting of the femoral head and increases the overall stability and anti‐rotation effect. In addition, the unique sliding compression mechanism of FNS allows the fracture ends to closely contact each other, promoting fracture healing.

In this study, femoral head necrosis did not occur in the FNS group but did occur in one patient in the cannulated screw group. Some studies have found that the stability of femoral neck fractures is of great significance for revascularization of the femoral head and plays an important role in promoting bone healing and in reducing the rate of femoral head necrosis^16^, ^17^. Moreover, it has been reported that a large implant volume may interfere with revascularization of the femoral head and increase the incidence of femoral head necrosis^18^. The diameters of the screw bolt and the anti‐rotation screw of the FNS in this study were 10 mm and 6.4 mm, respectively; thus, the volume of the FNS implant was significantly smaller than that of the three cannulated screws. In addition, the combination and smaller size of the son–mother nail can effectively reduce damage to the femoral head, and it is also beneficial for preserving bone mass in the femoral neck. Nevertheless, no significant difference in the incidence of femoral head necrosis was identified between the groups, which may be attributed to the small sample size and short follow‐up period.

### Limitations of the Study

Deficiencies in this retrospective analysis should not be neglected. First, the surgeon independently selected the implants, leading to potential selection bias. However, we adopted a 1:1 pairing method to eliminate the interference of many factors to the greatest extent, to reduce the level of individual variation, and to make the test group and the control group comparable. Second, because of the short time on the market and the high price of FNS, the accuracy of this study may be limited by a small sample size and short follow‐up period. Reasons for internal fixation failure and femoral head necrosis could not be definitively ascertained. In future studies, a large sample size and longer follow‐up are necessary to validate our findings.

### Conclusion

This study found that compared with cannulated screws, FNS is a suitable option for the treatment of Pauwels type‐3 femoral neck fractures. This approach is characterized by its accurate efficacy, simple procedure, reduced trauma, faster recovery, and fewer complications, though it is more expensive. FNS's excellent biomechanical performance and clinical efficacy make it a new choice for the treatment of unstable femoral neck fractures.

## Ethics Approval

This trial was approved by the Ethics Committee of Suzhou Municipal Hospital (IEC‐C‐008‐A07‐V1.0).

## Consent for Publication

Not application.

## Availability of Data and Materials

The dataset analyzed during the current study is not publicly available due to patient privacy but is available from the corresponding author on reasonable request
